# Supplementary material for: A catechol-O-methyltransferase genetic variant impacts functional movement in tactical athletes
Source: PLoS One. 2025 Feb 7;20(2):e0316127. doi: 10.1371/journal.pone.0316127 (PMC11805364; doi:10.1371/journal.pone.0316127)
Supplement: Attachment — Submitted filename: Response to Reviewers.docx [file pone.0316127.s001.docx]

**Reviewer #1:**

Based on my assessment of the manuscript:

1. Technical Soundness:

a) The manuscript is technically sound in terms of its experimental design and approach. The research addresses an important gap by investigating the influence of the COMT rs737865 variant on functional movement, as well as its interaction with trauma exposure, in a specialized population of military personnel.

b) The methodology is clearly described, including the use of the Functional Movement Screen (FMS), genotyping, and trauma exposure assessment. The choice of these methods is appropriate for the research objectives.

2. Data Support for Conclusions:

a) The data presented support the conclusions drawn by the authors. There is a clear correlation between the rs737865 variant and functional movement, as well as a significant interaction between trauma exposure and the genotype. The findings related to gene-environment interactions (involving the rs737865 variant and trauma) are compelling and consistent with the data.

b) The conclusions about how rs737865 could be used in injury risk stratification and strength and conditioning programs are reasonable based on the findings.

3. Statistical Analysis:

a) The statistical analysis appears to have been performed appropriately. The authors used general linear models to assess the effects of rs737865 on functional movement, as well as regression models for trauma exposure. Covariates were screened and tested in line with established statistical approaches.

b) The reported p-values and effect sizes (η2p) are within acceptable ranges, and the alpha threshold (0.05) is standard. However, the post-hoc power analysis indicates that the sample size might have been modest, which the authors acknowledge as a limitation. Although the power falls slightly below conventional standards, the primary findings are statistically significant (p = 0.03), which mitigates concerns of Type I error.

4. Data Availability:

a) The manuscript states that the data underlying the findings are confidential and part of U.S. Government property. This limits the public availability of data, which may be an issue depending on journal policies. However, the authors provide a contact method for data-sharing requests, which adheres to ethical requirements considering the sensitive nature of the sample (military personnel).

5. Clarity and Language:

a) The manuscript is written in clear, standard English, and the overall presentation is intelligible. The structure follows a logical progression, with clear sections for methods, results, and discussion. The scientific language is appropriate for a specialized audience.

Author Response: The authors wish to thank Reviewer #1 for their time and appreciate their detailed and thorough review of the manuscript.

**Reviewer #2:**

Title and Abstract:

1. The title is informative and specific, effectively encapsulating the primary focus on the impact of the COMT rs737865 genetic variant on functional mobility in tactical athletes.

2. The abstract offers a succinct summary; it could be improved by a more explicit expression of the clinical significance of the findings, particularly in the context of injury risk assessment and rehabilitation programs for tactical athletes.

Author Response: Thank you for the suggestion. We have included an additional description of the clinical implications of the findings in the context of injury risk assessment as well as in strength and conditioning programs. This additional explanation is brief because we want to avoid overstating the clinical significance of our novel findings. To that end, we outline our plan to replicate and extend our findings with larger sample sizes (page 16, lines 292-293 in the tracked changes manuscript).

Introduction:

1. The introduction effectively establishes the connection between functional movement (FM), injury risk, and genetic factors, with a particular emphasis on the COMT gene and previous research on the relationship between trauma and FM.

2. Additional profundity could be achieved by providing a more detailed explanation of the potential biological effects of genetic variants such as rs737865 on FM beyond dopamine signaling.

Author Response: Excellent point. We inserted a brief description of the role of COMT in other monoamine neurotransmitters, namely epinephrine and norepinephrine, particularly in the context of pain and mental health (page 4, lines 71-73 in the tracked changes manuscript). The connection to pain is especially germane (see Estevez-Lopez et al., 2022, reference #6), given that we expected and tested a mediating role of pain within our statistical models. Altogether, this broadens our perspective on COMT mechanisms of action, and helps to further strengthen the biological plausibility of our hypothesis tests, which are visualized in Figs 1 & 2.

3. The potential applications of the findings in personalized training programs for tactical athletes could be more explicitly highlighted in the introduction.

Author Response: Per our previous response, we inserted a more explicit description of the clinical implications of the findings in the context of injury risk assessment as well as in strength and conditioning programs in the abstract and introduction. However, given the novelty of our findings, and our current plans to replicate and extend with larger sample sizes, we are cautious not to overstate their clinical significance.

Methods

1. The methodology is comprehensive, with a particular emphasis on the recruitment of participants, genetic analysis, trauma exposure assessment, and FM evaluation.

2. Although the genotyping and FM assessment procedures are explicitly defined in the study, a flow diagram that delineates the study design from participant recruitment to data analysis could be beneficial in comprehending the study's structure.

Author Response: Thank you for the suggestion. We have re-reviewed the methods to ensure clarity for the reader. In our experience, flow diagrams are useful for complex, prospective/longitudinal studies, such as RCTs with multiple arms, intent-to-trait, and/or loss-to-follow up. Because this study design is cross-sectional, with very few measures, we don’t believe that a flow diagram is necessary. However, if the Reviewer and/or Editor feel strongly to the contrary, we will gladly produce one so long as we have room per the journal’s table/figure limitations.

3. The Functional Movement Screen (FMS) was selected over other FM assessments, and the study could benefit from a concise explanation of its alignment with the study's objectives.

Author Response: We have added that the FMS was selected based on its generalizability to the performance of EOD operators, as well as its known inter-rater and test-retest reliability (page 7, lines 153-154 in the tracked changes manuscript).

Results

1. The results are presented in a plain manner, demonstrating statistically significant associations between the rs737865 variant and FM scores.

2. The sample size is identified as a constraint, and further power calculations could verify this. To be more precise, a larger sample size could have enabled a more conclusive analysis of the rs737865 and FM interaction by enhancing statistical power.

Author Response: We have addressed the impact of a modest sample size and statistical power in our limitations paragraph (page 16, lines 287-295 in the tracked changes manuscript). Specifically, the modest sample size yielded statistical power that registered below conventional standards (e.g., 0.80); however, this was counterbalanced by the observed *p*-value of our primary hypothesis test (*p* = 0.03). This translates to a 3% likelihood of achieving a Type I error, which is the relevant error of concern in the case of null rejection (versus a Type II error).

3. Further exploration of this subgroup could provide a more comprehensive understanding of the findings, as rs737865 predominantly moderated the trauma-FM relationship in AA carriers.

Author Response: We concur that the AA subgroup warrants further exploration, which is a central goal for our follow-on studies with larger sample sizes. In the current study, the AA subgroup findings support the hypothesis that rs737865 moderated our previously published result, which linked psychological trauma exposure to FM (independent of physical injury).

Discussion

1. The findings are effectively contextualized within the broader context of genetics, FM, and trauma exposure in the discussion.

2. The study's findings on the interaction between rs737865 and trauma on FM are valuable; however, it would be beneficial to provide further detail on how these results could be generalized to other athletic or military populations.

Author Response: At this time, we cannot be sure precisely how these results will generalize. However, we acknowledged in the limitations section (page 16, line 292 in the tracked changes manuscript) that our follow-on studies will help to determine whether, or to what extent, the findings may generalize to other populations.

3. A discussion of potential gene-environment interactions in relation to other COMT variants (e.g., rs4680) could add substance, as rs737865, rs4680, and rs165599 frequently co-occur as a haplotype.

Author Response: We concur that the haplotype could be particularly informative. We are currently evaluating budgets to establish the feasibility of studying this haplotype in future studies.

4. The study could be improved by providing a more explicit statement regarding the specific impact of these insights on tactical training protocols, which would increase the practical relevance of the findings.

Author Response: Within the abstract (pages 2-3, lines 46-49 in the tracked changes manuscript) and the introduction (page 3, lines 62-65 in the tracked changes manuscript), we inserted a more explicit description of the clinical implications for our findings in the context of injury risk assessment as well as in strength and conditioning programs. As noted earlier, we are cautious not to overstate the clinical significance, given the novelty of the finding and our current plans to replicate and extend these results with larger sample sizes.

Overall recommendation

This manuscript offers new perspectives on the correlation between FM and the COMT rs737865 variant in tactical athletes. The results have significant implications for the development of personalized strategies for injury prevention and rehabilitation. This manuscript could be a valuable contribution to the field of sports genomics and precision training with minor revisions to improve the lucidity of the clinical implications and a more thorough examination of the genetic mechanisms involved.

Author Response: We thank the Reviewer for their comments and suggestions. We believe the manuscript is strengthened because of this feedback.

**Additional Editor Comments:**

The research examined the association between the COMT rs737865 variant and FM in specialized military personnel, taking into account the influence of trauma exposure. Identifying genetic susceptibility factors linked to FM may offer new insights into its underlying causes, enabling identifying individuals at higher risk for FM. The findings suggest a connection between the COMT gene and FM, although the precise mechanisms remain unclear.

Revisions needed:

1. Enhance the abstract to convey the clinical significance of the findings more clearly, particularly in relation to injury risk assessment and rehabilitation programs for tactical athletes.

Author Response: Completed, as requested by Reviewer #2.

2. Expand the introduction with a more detailed discussion of the potential biological impacts of genetic variants, like rs737865, on FM beyond their role in dopamine signaling.

Author Response: Completed, as requested by Reviewer #2. We acknowledged the effects of COMT metabolism on epinephrine and norepinephrine and focused the discussion on relationships with pain due to its germane role in our (mediational) hypothesis tests.

3. In the introduction, emphasize the practical applications of these findings in designing personalized training programs for tactical athletes.

Author Response: Completed, as requested by Reviewer #2.

4. Include additional detail on how these results might be generalized to other athletic or military populations.

Author Response: Completed, as requested by Reviewer #2.

This manuscript has the potential to be a valuable contribution to sports genomics and precision training. Minor revisions to clarify the clinical implications and provide a deeper exploration of genetic mechanisms will further enhance its impact.

Author Response: Completed, as requested by Reviewer #2, with the caveat that we are cautious not to overstate the potential implications of our findings.
